# Mission Critical, a Call to Action for Implementation of the Recommendations of the Canadian Pain Task Force

**DOI:** 10.1080/24740527.2024.2346253

**Published:** 2024-06-17

**Authors:** Mary Lynch

**Affiliations:** FRCPC, Founder (Pain Medicine) Royal College Physicians and Surgeons, Canada; Department of Anesthesia, Pain Management and Perioperative Medicine, Psychiatry and Pharmacology, Dalhousie University, Halifax, Nova Scotia, Canada

In this issue, Bosma et al.^1^ identify that there has been a significant increase the number of patients denied care at interdisciplinary pain treatment centers due to the complexity of mental health needs. A retrospective review of all declined referrals to the Toronto Academic Pain Medicine Institute (TAPMI) was conducted. TAPMI is a partnership between five academic pain centers in Toronto that, together, receive approximately 7000 referrals per year. They examined all declined referrals and compared the numbers and reasons for those not given an appointment in a prepandemic year (2018) versus a postpandemic year (2022). They found an increase from 11.43% to 18.39% of patients who were denied an appointment because of mental health complexity.

In a previous study, Dassieu and colleagues^2^ found that approximately one in four Canadian multidisciplinary pain treatment centers excluded patients if a co-occurring mental health disorder and/or a substance use disorder was present. Previous work has clearly identified that people living with chronic pain conditions who are on wait lists for pain care have a very poor quality of life, with 50% experiencing severe or extremely severe depression and 35% reporting suicidal ideation.^3^ The Canadian Pain Task Force (CPTF) explicitly mentions the importance of addressing people living with pain and mental illness as well as those living with chronic pain who use substances.^4^ In a survey of Canadian pain clinics during the early months of the pandemic, it was found that there was a substantial decrease in pain services with an escalation in pain, stress, and medication use among people with chronic pain.^5^

The current study confirmed that many people living with chronic pain have complex mental health needs, and this has become worse since the pandemic. The fact that one-quarter of Canadian pain treatment centers exclude patients with co-occurrent mental health disorders was already unacceptable. Bosma and colleagues^1^ have now identified that there has been an escalation in the number of people with pain and mental health issues who are being turned away. The situation was dire and is getting worse. The implementation of the recommendations of the CPTF is more important now than ever. We have reached the point of Mission Critical. This is a call to action for all people living with pain and those who care for and about them to advocate for urgent implementation of the CPTF recommendations.

## References

[1] Bosma R, Rosenbloom BN, Burke E, Aquino C, Stanley C, Coombs K, Adriano N, Shamalla J, Clarke H, Flamer D, et al. An examination of referrals declined for chronic pain treatment: there is increasing mental health complexity within treatment seeking patients with chronic pain over time. Can J Pain. 2024. *this issue*. doi:10.1080/24740527.2024.2337074. PMC11210908 38938328

[2] Dassieu L, Choinière M, Saint-Jean L, Webster F, Peng P, Buckley N, Gilron I, Williamson O, Finley GA, Baerg K, et al. Frequency and characteristics of patient exclusion criteria in Canadian multidisciplinary pain treatment facilities: a cross-sectional study. Can J Anesth/J Can Anesth. 2022;69(7):849–1. doi:10.1007/s12630. 35304693

[3] Choiniere M, Dion D, Peng P, Banner R, Barton P, Boulanger A, Clark AJ, Gordon A, Guerriere D, Geurtin MC, et al. The Canadian STOP-PAIN project-part 1: who are the patients on the waitlists of multidisciplinary pain treatment facilities? Can J Anesth. 2010;57(6):539–48. doi:10.1007/s12630-010-9305-5. 20393821

[4] https://www.canada.ca/en/health-canada/corporate/about-health-canada/public-engagement/external-advisory-bodies/canadian-pain-task-force/report-2021.html.

[5] Lynch ME, Williamson OD, Banfield JC. COVID-19 impact and response by Canadian pain clinics: a national survey of adult pain clinics. Can J Pain. 2020;4(1):204–09. doi:10.1080/24740527.2020.1783218. 33987499 PMC7951169

